# HIF‐1α knockdown attenuates inflammation and oxidative stress in ischemic stroke male rats via CXCR4/NF‐κB pathway

**DOI:** 10.1002/brb3.70039

**Published:** 2024-09-18

**Authors:** Gao Chen, Xi Wang, Zhan Jin, Gao‐Bo Hu, Qi‐Hui Yu, Hai‐Yan Jiang

**Affiliations:** ^1^ School of Medicine Quzhou College of Technology Quzhou Zhejiang China; ^2^ Department of Urology The Quzhou Affiliated Hospital of Wenzhou Medical University, Quzhou People's Hospital Quzhou Zhejiang China; ^3^ Department of Gynecology Quzhou Maternal and Child Health Care Hospital Quzhou Zhejiang China

**Keywords:** HIF‐1α/CXCR4/NF‐κB axis, inflammation, ischemic stroke, oxidative stress

## Abstract

**Background:**

Hypoxia inducible factor‐1α (HIF‐1α) is a sensitive indicator of oxygen homeostasis, of which the expression elevates following hypoxia/ischemia. This study reveals the specific mechanisms underlying the effects of HIF‐1α on ischemic stroke (IS).

**Methods:**

IS model was established using middle cerebral artery occlusion (MCAO)‐modeled male rats and oxygen glucose deprivation/reoxygenation (OGD/R)‐treated mice hippocampal cells HT22, followed by the silencing of HIF‐1α and the overexpression of C‐X‐C motif chemokine receptor 4 (CXCR4) and nuclear factor‐kappa B (NF‐κB). Following the surgery, Garcia's grading scale was applied for neurological evaluation. Cerebral infarcts and injuries were visualized using 2,3,5‐triphenyltetrazolium chloride and hematoxylin‐eosin staining. The levels of tumor necrosis factor‐α, Interleukin (IL)‐6, IL‐1β, malondialdehyde, and 8‐hydroxy‐2′‐deoxyguanosine, were calculated via ELISA. MTT assay and lactate dehydrogenase (LDH) assay kit were adopted to determine the viability and cytotoxicity of OGD/R‐modeled cells. Reactive oxygen species (ROS) generation was evaluated using a 2′‐7′dichlorofluorescin diacetate (DCFH‐DA) probe. The levels of HIF‐1α, CXCR4, and NF‐κB p65 were quantified via Western blot and immunofluorescence, respectively.

**Results:**

HIF‐1α knockdown improved Garcia's score, attenuated the cerebral infarct, inflammation, and ROS generation, and alleviated the levels of inflammatory cytokines and CXCR4/NF‐κB p65 in MCAO‐modeled rats. Such effects were reversed following the overexpression of CXCR4 and NF‐κB. Also, in OGD/R‐treated HT22 cells, HIF‐1α silencing diminished the cytotoxicity and ROS production and reduced the expressions of CXCR4/NF‐κB p65, while promoting viability. However, CXCR4/NF‐κB p65 overexpression did the opposite.

**Conclusion:**

HIF‐1α knockdown alleviates inflammation and oxidative stress in IS through the CXCR4/NF‐κB pathway.

## Graphical abstract

In this study, HIF‐1α‐specific short hairpin RNA is used to silence HIF‐1α. HIF‐1α silencing attenuates inflammation and oxidative stress in ischemic stroke male rats and oxygen‐glucose deprivation/reoxygenation (OGD/R) mice hippocampal cells via the CXCR4/NF‐κB pathway.

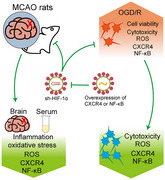

## 1 INTRODUCTION

Ischemic stroke (IS) is currently one of the most significant causes accounting for both neurological morbidity and mortality worldwide, and is caused primarily by interrupted cerebral blood flow, with an annual mortality of approximately 5.5 million (Maida et al., 2020; Paul & Candelario‐Jalil, 2021; Qin et al., 2022). The primary prevention strategies for IS include the modification of lifestyles, diet, and the treatment of relevant risk factors (Diener & Hankey, 2020). Also, timely revascularization therapies have been already proven as effective treatments in the early stage of IS; however, they have some limitations as well, like the short therapeutic window, rigorous criteria of eligibility and the contradictions (Su et al., 2020). Thus, the therapeutic options for IS warrant further investigation.

Accumulating studies have laid great emphasis on inflammation and oxidative stress in IS (DeLong et al., 2022; Orellana‐Urzúa et al., 2020). As for the mechanisms underlying inflammation‐driven damage IS, increasing research has highlighted the close association between excessive reactive oxygen species (ROS) generation and stroke‐induced inflammatory response (Candelario‐Jalil et al., 2022). Meanwhile, some dying cells can release some molecules contributing to the activation of surrounding viable tissue or infiltrating cells, which, through different signaling cascades, ultimately triggers the proinflammatory intracellular signaling cascades and transcription factors like nuclear factor‐kappa B (NF‐κB) and the release of proinflammatory cytokines, interleukin‐6 (IL‐6), interleukin‐1β (IL‐1β), and tumor necrosis factor‐α (TNF‐α), for instance (Gülke et al., 2018). Therefore, it remains a question of how to modulate the post‐IS inflammatory response.

The profound contribution of genetic causes in the genesis of IS has been already acknowledged (Ekkert et al., 2021). Of note, hypoxia inducible factor‐1α (HIF‐1α), an oxygen‐sensitive subunit of the heterodimeric transcription factor HIF‐1, has caught the attention of scholars both at home and abroad (Zhang, Yao et al., 2018). As a sensitive oxygen homeostasis regulator, HIF‐1α plays an active role in diverse processes concerning the pathophysiology of stroke, with its expression elevated following hypoxia/ischemia, which thus makes it of great importance to reveal the specific role and therapeutic targets for IS (He et al., 2021). Some opposing results have been concluded based on the current investigation of HIF‐1α, as shown in the results that HIF‐1α can either act as an oxygen stability transcription regulator and a main adaptive response stimulant or the stimulator of proapoptotic molecules (Amalia et al., 2020). When it comes to the specific mechanisms, it has been highlighted that HIF‐1α could target the stromal cell‐derived factor‐1 (SDF‐1)/C‐X‐C motif chemokine receptor 4 (CXCR4) pathway, which makes the extension of ischemic vessels toward the areas with sufficient blood supply (Huang et al., 2018; Young et al., 2009). Another study by Liu et al. (2023) has suggested the modulatory effects of CXCR4 on pyroptosis and lipid peroxidation in postsubarachnoid hemorrhage (SAH) early brain injury via regulating NF‐κB (which has been revealed to be positively regulated by HIF‐1α as well) (D'Ignazio et al., 2020). Accordingly, it has become the aim of our current study to confirm the involvement and effects of HIF‐1α/CXCR4/NF‐κB in IS, with the hope of providing novel interventional and therapeutic strategies for IS.

## 2 MATERIAL AND METHODS

### 2.1 Ethics statement

All animal experiments were implemented complying with the Chinese National Research Council Guidance for the Care and Use of Laboratory Animals and permitted by the Animal Experimentation Ethics Committee of Zhejiang Eyong Pharmaceutical R&D Center (license no. SYXK (Zhejiang) 2021‐0033) (Approval resolution number: ZJEY‐20230622‐01).

### 2.2 Animals

Specific pathogen‐free male Sprague‐Dawley rats (250–270 g, *n* = 36) were available from Shanghai Jihui Laboratory Animal Care Co., Ltd. (license no. SCXK (Shanghai) 2022‐0009) and reared at the temperature of 20–26°C and the humidity of 50–60% with a 12‐h day/night cycle and free access to food and water.

For subsequent assays, all rats were allocated to the following groups as needed, with 6 in each group: Sham, I/R, I/R+sh‐NC, I/R+sh‐HIF‐1α, I/R+sh‐HIF‐1α+oe‐CXCR4, and I/R+sh‐HIF‐1α+oe‐NF‐κB. The detailed processing of these rats is illustrated below.

### 2.3 Stereotactic surgery

Prior to the surgery, the adenoviruses containing the control short hairpin RNA (sh‐NC, 3.16 × 10^10^ pfu/mL), HIF‐1α‐specific short hairpin RNA (sh‐HIF‐1α, 2.51 × 10^10^ pfu/mL), CXCR4‐specific overexpression plasmid (oe‐CXCR4, 2.51 × 10^10^ pfu/mL), and NF‐κB‐specific overexpression plasmid (oe‐NF‐κB, 2.51 × 10^10^ pfu/mL) were all obtained from Shanghai GenePharma Co., Ltd.

The stereotactic surgery was implemented as per the guidance of Wang et al. (2013). Following the anesthesia via 2% isoflurane (PHR2874, Sigma, USA), the rats were put on the stereotaxic instrument in a supine position and disinfected. The skin around the skull was shaved to expose the bregma. The fascia was rinsed with 3% hydrogen peroxide to remove any excessive blood.

For rats in the Sham and I/R groups, only an equivalent volume of physiological saline was given. For the remaining four groups, the bregma of these rats was set as the stereotaxic zero and the adenoviruses were injected to 2 selected sites at the required coordinates: (a) 1 mm rostral to the bregma, 2 mm lateral to the midline, and 1.2 mm vertical to the dura and (b) 3 mm caudal to the bregma, 1.5 mm lateral to the midline, and 1.2 mm to the dura. The rate and volume of these lentiviruses were set to 0.3 µL/min and 2 µL for each site. The microinjector was kept for 5 min at the end of injection before withdrawal, and the skin was sutured. The rats were placed on the heating pad (37°C) until resuscitation.

### 2.4 Animal modeling

Following a week post stereotactic surgery, the animal model of middle cerebral artery occlusion (MCAO) with reperfusion was applied to mimic IS as per an existing article (Zhang et al., 2019). In detail, all rats were anesthetized via 5% isoflurane for anesthesia and 2% isoflurane for maintain, a midline neck incision was created. The left common carotid artery (CCA), internal carotid artery (ICA) and external carotid artery (ECA) were separated as needed, followed by the insertion of a 40‐mm‐length nylon suture (diameter: 0.236 mm) from the CCA to ICA and to the middle cerebral artery (MCA) to mimic ischemia. The suture was withdrawn after the occlusion for 2 h to allow the 24‐h reperfusion. Rats in the sham group received the same procedure without the occlusion of MCA. The rectal temperature of rats was maintained at 37 ± 0.2°C using an electronic heating mat.

### 2.5 Neurological score evaluation

The neurological score of rats in each group (*n* = 6 each group) was evaluated on days 1 and 3 post the modeling by an investigator blinded to the allocation via the Garcia 18‐point scoring system (Garcia et al., 1995). The system comprises six tests: (1) spontaneous activity, (2) symmetry in the movement of four limbs, (3) forepaw outstretching, (4) climbing, (5) body proprioception, and (6) response to vibrissae touch. Higher scores indicate better performance.

### 2.6 Sample collection

All rats were finally sacrificed via inhalation of CO_2_ following the evaluation of neurological scores. The blood was then obtained from the heart and the brain tissue was harvested and stored in liquid nitrogen until use.

### 2.7 2,3,5‐triphenyltetrazolium chloride (TTC) staining

TTC staining was applied to assess the cerebral infarct volume as needed (Lv et al., 2017). Specifically, the brain tissues (*n* = 3 each group) were weighted and sectioned coronally into the slices with a thickness of 2 mm. Subsequently, the slices were incubated in 1% TTC staining solution (F603BA0025, Sangon, China) at 37°C for 15 min and fixed in 4% fixative paraformaldehyde for 30 min. The results were photographed and analyzed in ImageJ (v. 1.51, National Institutes of Health, USA). The infarct rate was calculated as per the formula:

$$\mathrm{Cerebral}\;\mathrm{Infarct}\;\mathrm{Rate}\;\;\left(\%\right)=\frac{\mathrm{total}\;\mathrm{area}-\mathrm{red}\;\mathrm{area}}{\mathrm{total}\;\mathrm{area}\;}\;\;\times 100\%.$$

### 2.8 Hematoxylin‐eosin (HE) staining

The prepared brain tissue (thickness: 5 µm) (*n* = 3 each group) was dewaxed in xylene (10023418, Sinopharm Chemical Reagent Co., Ltd, China) and rinsed in graded ethanol (100092683, Sinopharm Chemical Reagent Co., Ltd, China). After the washing, the tissue was dyed with hematoxylin (H3136, Sigma) for 5 min, rinsed with distilled water, and immersed in a differentiation buffer for 30 s. Following the rinse in distilled water again, the sections were stained with eosin solution (E4009, Sigma) and soaked in the distilled water. Finally, the tissues were dehydrated in ethanol, transparentized in xylene, and sealed in neutral gum (10004160, Sinopharm Chemical Reagent Co., Ltd, China). An optical microscope (Eclipse Ci‐L, Nikon, Japan) was applied to observe the results at a magnification of 200 times (Zhang, Liu et al., 2018).

### 2.9 ELISA

ELISA kits for TNF‐α (RX302058R, Ruixin Biotech, China), IL‐6 (RX302856R, Ruixin Biotech, China), IL‐1β (RX302869R, Ruixin Biotech, China), malondialdehyde (MDA, Jiancheng Bio, Nanjing, China), and 8‐hydroxy‐2′‐deoxyguanosine (8‐OHdG, ab285254, Abcam, UK) were all obtained from the producers to measure the levels in both brain and serum of rats (*n* = 6 each group) as per the manuals. A microplate reader was applied to read at 450 nm.

### 2.10 Cell culture and grouping

Mice hippocampal neuron cell line HT22 (iCell‐m020, iCell Bioscience, China) was grown in Dulbecco's modified Eagle's Medium (FI101‐01, Transgen Biotech, China) with the supplementation of 10% bovine calf serum (FS301‐02, Transgen Biotech, China) and 1% penicillin‐streptomycin (FG101‐01, Transgen Biotech, China) and incubated in the CO_2_ incubator (BB150, Thermo, USA) at 37°C with 5% CO_2_.

In vitro ischemia was mimicked by OGD/R modeling in HT22 cells (Li et al., 2023). HT22 cells were seeded into a T25 culture bottle with 1 × 10^5^ cells. After cells growing to 80% confluence, the normal culture medium for HT22 cells (supplemented with fetal bovine serum and glucose) was discarded and replaced with the one only containing the medium, and the cells were placed in the incubator with 5% CO_2_, 1% O_2,_ and 94 N_2_. Following the culture for 2 h, the cells continued to grow in the normal culture medium and returned to the normal 5% CO_2_ incubator (95% air) for 24‐h culture.

### 2.11 MTT assay

HT22 cells in the logarithmic phase were digested in 0.25% trypsin (T1350, Solarbio, China) and the density was adjusted to 5 × 10^3^ cells/well in 96‐well plates. 20 µL MTT solution (5 mg/mL, ST316, Beyotime, China) was added to these cells for an additional 4‐h culture. Following the centrifugation at 1500 rpm for 10 min, the culture medium was carefully sucked, and 100 µL DMSO was added to each well to dissolve the crystal formed. After a gentle shake for 20 min, the absorbance was recorded in a microplate reader (CMaxPlus, Molecular Devices, LLC., USA) at 490 nm. The viability of cells in each group (*n* = 6) was calculated based on the recording.

### 2.12 Lactate dehydrogenase (LDH) quantification test

HT22 cells were seeded into the 96‐well plates at the density of 4 × 10^4^ cells/mL (*n* = 6 per group) and the LDH generated was quantified using a commercial assay kit (C0016, Beyotime, China) according to the protocols. The microplate reader was applied to read the absorbance at 490 nm and the cytotoxicity (%) was accordingly quantified.

### 2.13 Immunofluorescence

Immunofluorescence assay was divided into two parts in our current study.

The first part mainly dealt with the quantification of ROS generated. For the quantification of ROS contents of rats in vivo, the brain tissues (*n* = 3 per group) were homogenized on ice containing 1 mM ethylene diamine tetraacetic acid and 50 mM sodium phosphate buffer and added with 25 mM 2′−7′dichlorofluorescin diacetate (DCFH‐DA) provided by the assay kit (S0033S, Beyotime, China) for 30‐min incubation at 37°C in the dark. Following the staining of nuclei via 4′,6‐diamidino‐2‐phenylindole (DAPI), the fluorescence values were determined in a fluorescence microscope (Eclipse C1, Nikon, Japan) at the magnification of 200 times. As to the measurement of ROS in HT22 cells in vitro, HT22 cells (*n* = 3 per group) in the logarithmic phase were digested for the preparation of cell suspension (1 × 10^5^ cells/mL) and 1 mL cell suspension was added to the 12‐well plates. Hereafter, when cells became 70–80% confluent, the diluted DCFH‐DA probe was added to each well for 20‐min incubation. These cells were then rinsed in serum‐free medium, fixed in 4% fixative for 10 min, and transparentized in 0.5% Triton X‐100 for 2 min. Following the completion of rinsing, the cells were sealed and observed in the fluorescence microscope at the magnification of 200 and 400 times.

The second part aims to quantify the levels of HIF‐1α/CXCR4/NF‐κB p65 in different groups of HT22 cells. As described above, HT22 cells (*n* = 3 per group) in the 12‐well plates were fixed in 4% fixative and transparentized using 0.5% Triton X‐100, followed by being blocked in 3% bovine serum albumin (BSA) and incubated with the primary antibodies (Abcam, UK) against HIF‐1α (ab1, 1:200), CXCR4 (ab181020, 1:500), and NF‐κB p65 (ab207297, 1:500) at 4°C overnight. After the rinse using PBS thrice (5 min for each time), Alexa Flour 594‐labeled secondary antibodies (Abcam, UK) goat anti‐mouse IgG H&L (ab150116, 1:500) and goat anti‐rabbit IgG H&L (ab150086, 1:500) were added to incubate with these cells at room temperature for 1 h. DAPI was adopted to stain the nuclei and the cells were observed in the fluorescence microscope at magnification of 200 and 400 times.

### 2.14 Western blot

100 mg brain tissue sample (*n* = 3) was added with 1 mL ice‐cold RIPA lysis buffer (P0013B, Beyotime, China) and the protein concentration was quantified using the BCA working solution (PC0020, Beyotime, China). Then, the protein samples were separated via SDS‐PAGE, transferred to the PVDF membranes, and blocked with 5% skimmed milk. Hereafter, the membranes were incubated with the primary antibodies at 4°C overnight and the secondary antibody at room temperature for 1 h. The ECL system (610020‐9Q, Clinx Science Instruments Co., Ltd, China) was applied to visualize the membrane and the results were analyzed in ImageJ. The information on antibodies used is listed in Table 1.

**TABLE 1:** Information of antibodies.

Antibodies	Host	Catalog number	Dilution ratio	Producer	RRID
HIF‐1α antibody	Rabbit	AF1009	1:1000	Affinity	AB_2835328
CXCR4 antibody	Rabbit	ab181020	1:1000	Abcam	AB_2910168
p‐NF‐κB p65 antibody	Rabbit	AF2006	1:1000	Affinity	AB_2834435
NF‐κB p65 antibody	Rabbit	AF5006	1:1000	Affinity	AB_2834847
GAPDH antibody	Rabbit	10494‐1‐AP	1:10,000	Proteintech	AB_2263076
HRP‐linked antibody	Goat	7074	1:6000	Cell Signaling Technology	AB_2099233

RRID: research resource identifier; HIF‐1α: hypoxia inducible factor‐1α; CXCR4: C‐X‐C motif chemokine receptor 4; P‐NF‐κB p65: phosphorylated‐NF‐κB p65; NF‐κB p65: nuclear factor‐kappa B p65.

### 2.15 Statistical analyses

All experiments were done at least three times with at least triplicates in each group. All data were analyzed in SPSS 16.0 and expressed as means ± standard deviation. Normality testing was performed by the Shapiro–Wilk test. One‐way ANOVA followed by Tukey's test (for data follow a normal distribution) and Kruskal–Wallis *H* test (for data do not follow a normal distribution) were applied during the statistical analyses. The threshold of statistical significance was set when the *p*‐value was below .05.

## 3 RESULTS

### 3.1 Effects of HIF‐1α silencing on the cerebral infarct and injury in MCAO‐modeled rats

**FIGURE 1:**
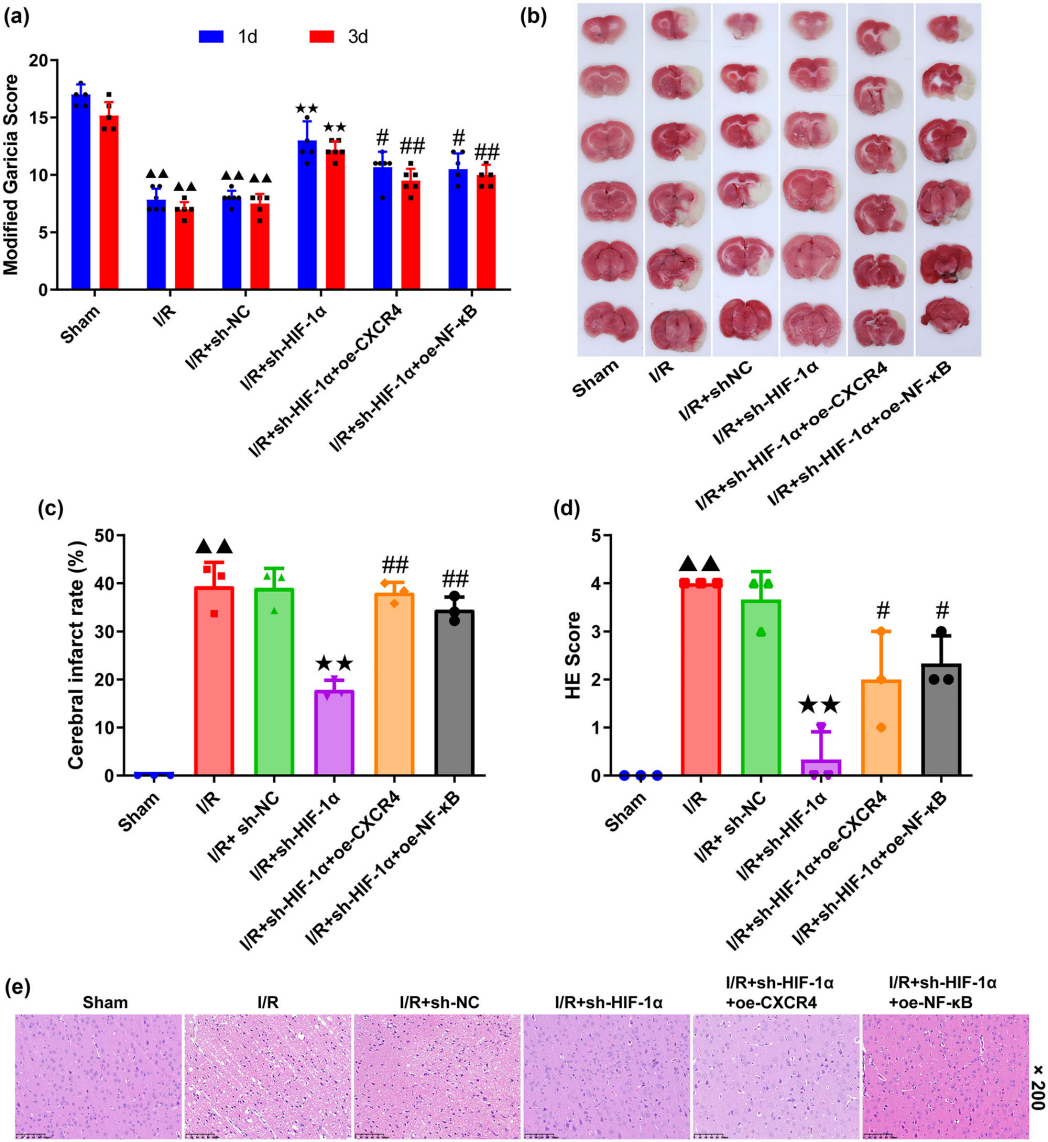
**HIF‐1α knockdown alleviates cerebral damage in MCAO‐modeled rats by targeting CXCR4/NF‐κB axis. (a)** Modified Garcia Score of rats in each group on day 1 and day 3 (*n* = 6). **(b, c)** Representative images of brain tissues of rats in each group based on TTC staining **(b)** and the quantified Cerebral Infarct Rate (%) (*n* = 3) **(c)**. **(d, e)** Quantified HE Score **(d)** and the symbolic pathology images of brain tissues of rats in the groups according to HE staining (*n* = 3) **(e)**. Magnification: ×200, scale bar: 100 µm. Data were presented as the mean ± standard deviation. ^▲▲^ *p *< .01, vs. Sham. ^★★^ *p *< .01, vs. I/R+sh‐NC. ^#^ *p *< .05, ^#^ *^#^p *< .01, vs. I/R+sh‐HIF‐1α. 1d: day 1; 3d: day 3; HIF‐1α: hypoxia inducible factor 1 subunit alpha; MCAO: middle cerebral artery occlusion; I/R: ischemia/reperfusion; sh‐RNA: short hairpin RNA; NC: negative control; oe‐CXCR4: overexpression plasmid of CXCR4; CXCR4: C‐X‐C motif chemokine receptor 4; NF‐κB: nuclear factor‐kappa B; TTC: 2,3,5‐triphenyltetrazolium chloride; HE: hematoxylin‐eosin.

The modified Garcia score (Figure 1a) was applied for neurological evaluation and all were in line with normal distribution, so a one‐way ANOVA was used to analyze the data (day 1: df = 5, *F* = 48.824; day 3: df = 5, *F* = 77.114; both *p *= .000). Tukey's test as the post hoc test after ANOVA was used. On both days 1 and 3, a reduced score was observed in MCAO‐modeled rats (I/R and I/R+shNC group compared to sham group, days 1 and 3: *p *= .000) (Figure 1a), whereas the silencing of HIF‐1α led to an increase (I/R+sh‐HIF‐1α group compared to I/R+shNC group, days 1 and 3: *p *= .000) (Figure 1a). Further, the score was decreased after the overexpression of both CXCR4 and NF‐κB in HIF‐1α‐silenced MCAO‐modeled rats (I/R+sh‐HIF‐1α+oe‐CXCR4 and I/R+sh‐HIF‐1α+oe‐NF‐κB group compared to I/R+sh‐HIF‐1α group: *p *= .024/.013 at day 1; *p *= .000/.002 at day 3) (Figure 1a). Based on TTC staining results, the cerebral infarct rates were counted and follow an approximate normal distribution (Figure 1b and c). A one‐way ANOVA was used to analyze TTC results (df = 5, *F* = 82.484, *p *= .000) MCAO processing evidently aggravated the cerebral infarct (I/R and I/R+shNC group compared to sham group: *p *= .000) (Figure 1b and c) yet HIF‐1α silencing caused a visible decrease in the infarct rate (I/R+sh‐HIF‐1α group compared to I/R+shNC group: *p *= .000) (Figure 1b and c). Nevertheless, the infract rate was increased following CXCR4 and NF‐κB overexpression in HIF‐1α‐silenced MCAO‐modeled rats (I/R+sh‐HIF‐1α+oe‐CXCR4 and I/R+sh‐HIF‐1α+oe‐NF‐κB groups compared to I/R+sh‐HIF‐1α group: both *p *= .000) (Figure 1b and c).

The results from H&E staining are available in Figure 1d and e and follow an approximate normal distribution. A one‐way ANOVA was used to analyze the scores (df = 5, *F* = 24.567, *p *= .000) followed using Tukey's test. Basically normal brain tissue could be observed in rats of the Sham group. Oppositely, large number of crumpled neurons and severe cerebral injury were seen in rats of both I/R and I/R+sh‐NC groups, with an increased HE Score (compared to sham group, both *p *= .000) (Figure 1d). Meanwhile, the number of crumpled neurons was fewer and the damage was alleviated in rats of the I/R+sh‐HIF‐1α group (compared to I/R+shNC group, *p *= .000) (Figure 1d), whereas rats of the last two groups demonstrated more crumpled neurons and vacuolar lesions in comparison with those of I/R+sh‐HIF‐1α group (I/R+sh‐HIF‐1α+oe‐CXCR4 and I/R+sh‐HIF‐1α+oe‐NF‐κB groups compared to I/R+sh‐HIF‐1α group: *p *= .037/.011) (Figure 1d).

### 3.2 Effects of HIF‐1α knockdown on the proinflammatory indicators of MCAO‐modeled rats

**FIGURE 2:**
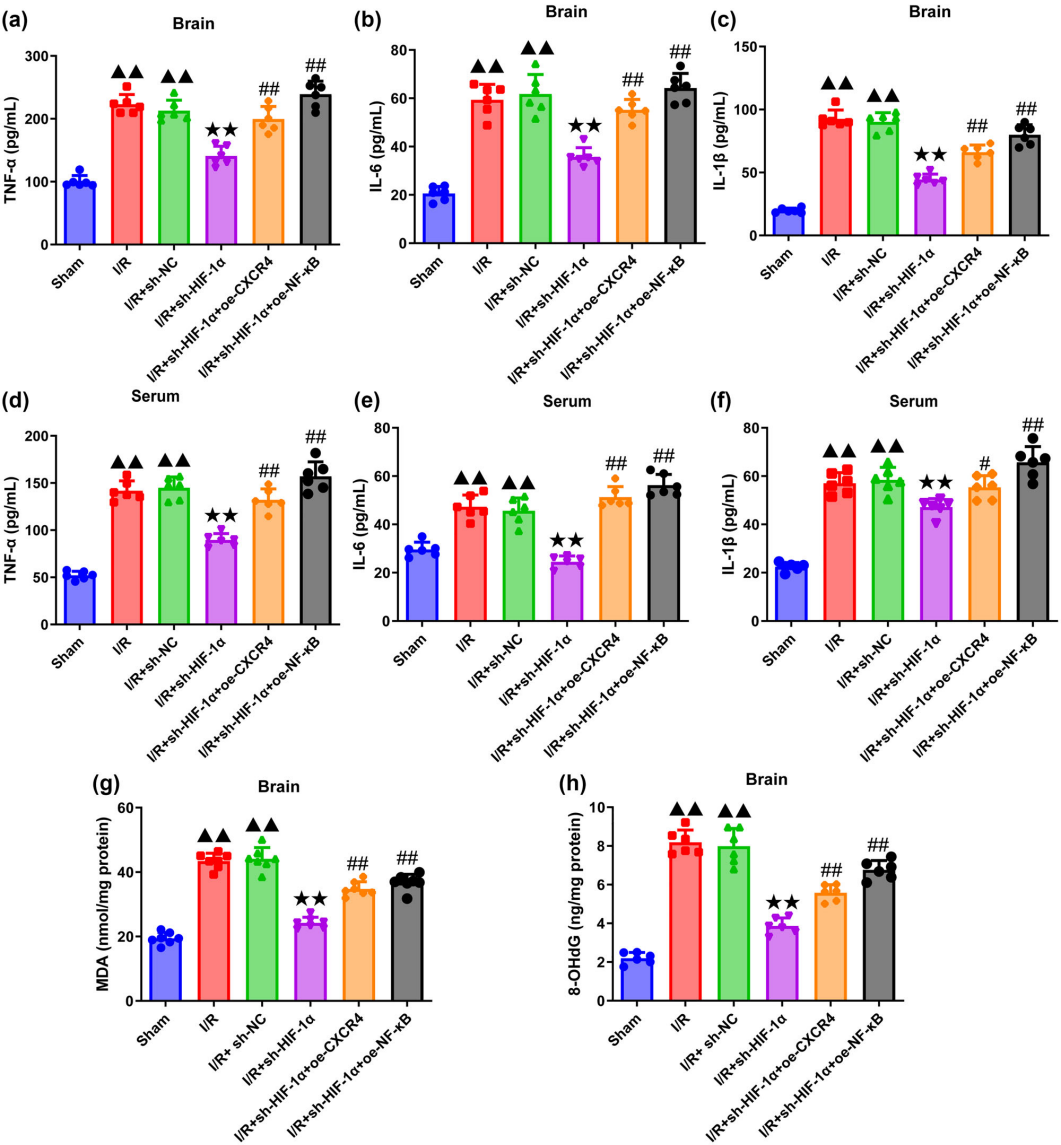
**HIF‐1α silencing attenuates cerebral inflammation in MCAO‐modeled rats via targeting CXCR4/NF‐κB axis. (a–c)** ELISA was applied to quantify the levels of TNF‐α **(a)**, IL‐6 **(b),** and IL‐1β **(c)** in the brain tissue of rats in each group (*n *= 6). **(d–f)** The levels of TNF‐α **(d)**, IL‐6 **(e),** and IL‐1β **(f)** in the serum sample of rats in each group were determined via ELISA (*n* = 6). **(g, h)** The content of MDA **(g)** and 8‐OHdG **(h)** in the brain tissue of rats in each group was calculated in ELISA (*n* = 6). Data were presented as the mean ± standard deviation. ^▲▲^ *p *< .01, vs. Sham. ^★★^ *p *< .01, vs. I/R+sh‐NC. ^#^ *p *< .05, ^#^ *^#^p *< .01, vs. I/R+sh‐HIF‐1α. TNF‐α: tumor necrosis factor‐α; IL‐6: interleukin‐6; IL‐1β: interleukin‐1β; MDA: malondialdehyde; 8‐OHdG: 8‐hydroxy‐2′ ‐deoxyguanosine.

The quantification results on the proinflammatory indicators of rats in each group are displayed in Figure 2. All were in line with normal distribution, so one‐way ANOVA was used to analyze proinflammatory indicator levels (All df = 5 and *p *= .000, TNF‐α, IL‐6, IL‐1β, MDA, and 8‐OHdG in brain: *F* = 62.225/59.483/136.369/84.862/103.560; TNF‐α, IL‐6, and IL‐1β in serum: *F* = 85.491/49.741/64.537) followed using Tukey's test. It was clear that the modeling of MCAO elevated the contents of TNF‐α, IL‐6, and IL‐1β in both brain and serum, with the increased cerebral MDA and 8‐OHdG content (compared to sham group, all *p *= .000) (Figure 2). Silencing of HIF‐1α, oppositely, diminished the contents of these indicators (compared to I/R+shNC group, TNF‐α, IL‐6, IL‐1β, MDA, and 8‐OHdG in brain: All *p *= .000; TNF‐α, IL‐6, and IL‐1β in serum: *p *= .000/.000/.002) (Figure 2), while such effect was reversed following the overexpression of both CXCR4 and NF‐κB (compared to I/R+sh‐HIF‐1α group, TNF‐α, IL‐6, IL‐1β, MDA, and 8‐OHdG in brain: All *p *= .000; TNF‐α, IL‐6, and IL‐1β in serum: All *p *= .000) (Figure 2).

### 3.3 Effects of HIF‐1α silencing on the ROS production in MCAO‐modeled rats

**FIGURE 3:**
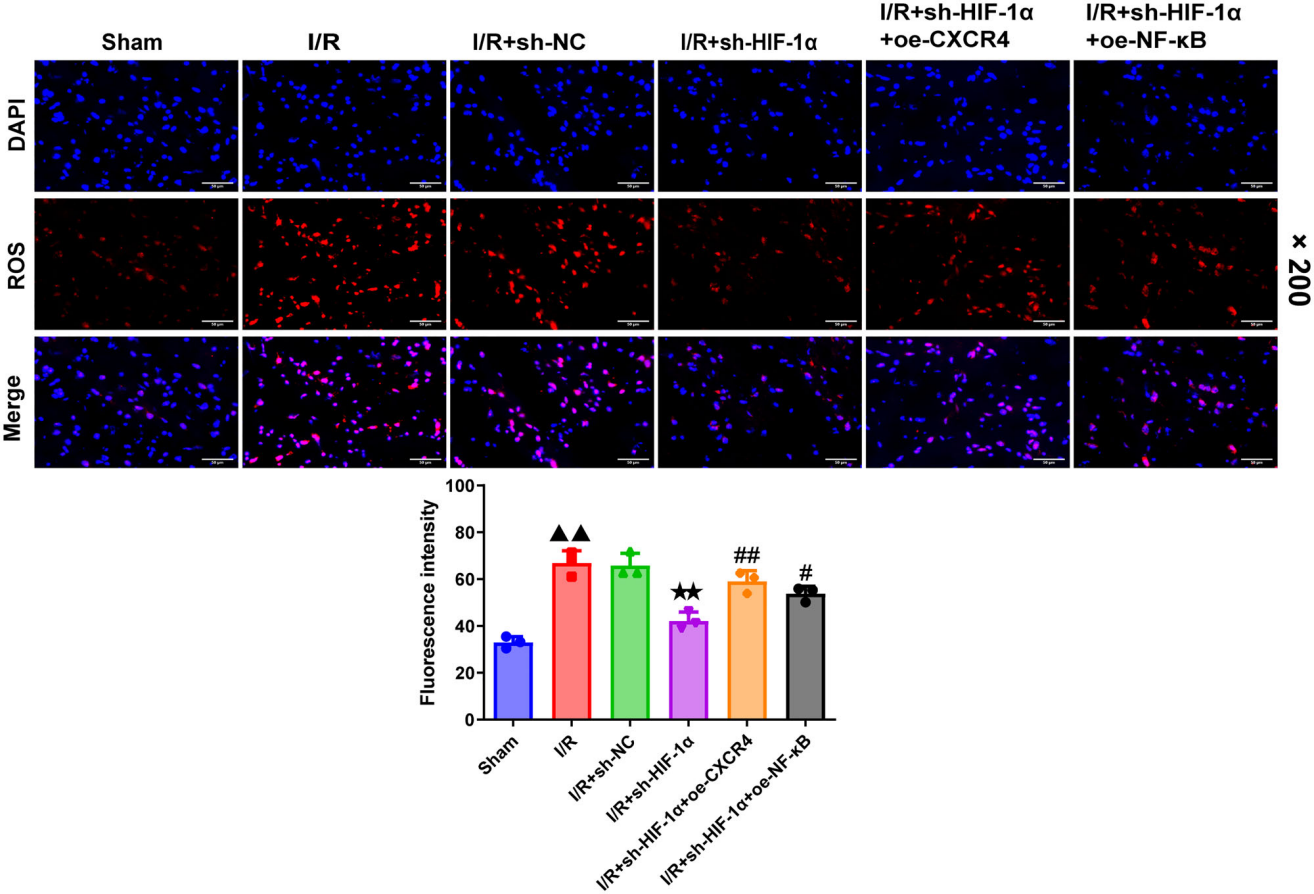
**HIF‐1α knockdown diminishes ROS generation in MCAO‐modeled rats via targeting CXCR4/NF‐κB axis**. Immunofluorescence was applied to quantify the generation of ROS in each group of rats (*n* = 6). Magnification: ×200, scale bar: 50 µm. Data were presented as the mean ± standard deviation. ^▲▲^ *p *< .01, vs. Sham. ^★★^ *p *< .01, vs. I/R+sh‐NC. ^#^ *p *< .05, ^#^ *^#^p *< .01, vs. I/R+sh‐HIF‐1α. ROS: reactive oxygen species; DAPI: 4′,6‐diamidino‐2‐phenylindole.

A one‐way ANOVA was used to analyze ROS fluorescence intensity data (df = 5, *F* = 30.472, *p *= .000), followed by Tukey's test. MCAO modeling caused an upward trend in the ROS production in rats (compared to sham group, I/R group: *p *= .000) (Figure 3), while HIF‐1α silencing did the opposite (compared to I/R+sh‐NC group, I/R+sh‐HIF‐1α group: *p *= .000) (Figure 3, *p *< .01). Additionally, overexpression of CXCR4 and NF‐κB was confirmed to reverse the effects of HIF‐1α silencing on ROS production in these modeled rats (I/R+sh‐HIF‐1α+oe‐CXCR4 and I/R+sh‐HIF‐1α+oe‐NF‐κB groups compared to I/R+sh‐HIF‐1α group: *p *= .004/.05) (Figure 3).

### 3.4 Effects of HIF‐1α silencing on HIF‐1α/CXCR4/NF‐κB p65 expression in MCAO‐modeled rats

**FIGURE 4:**
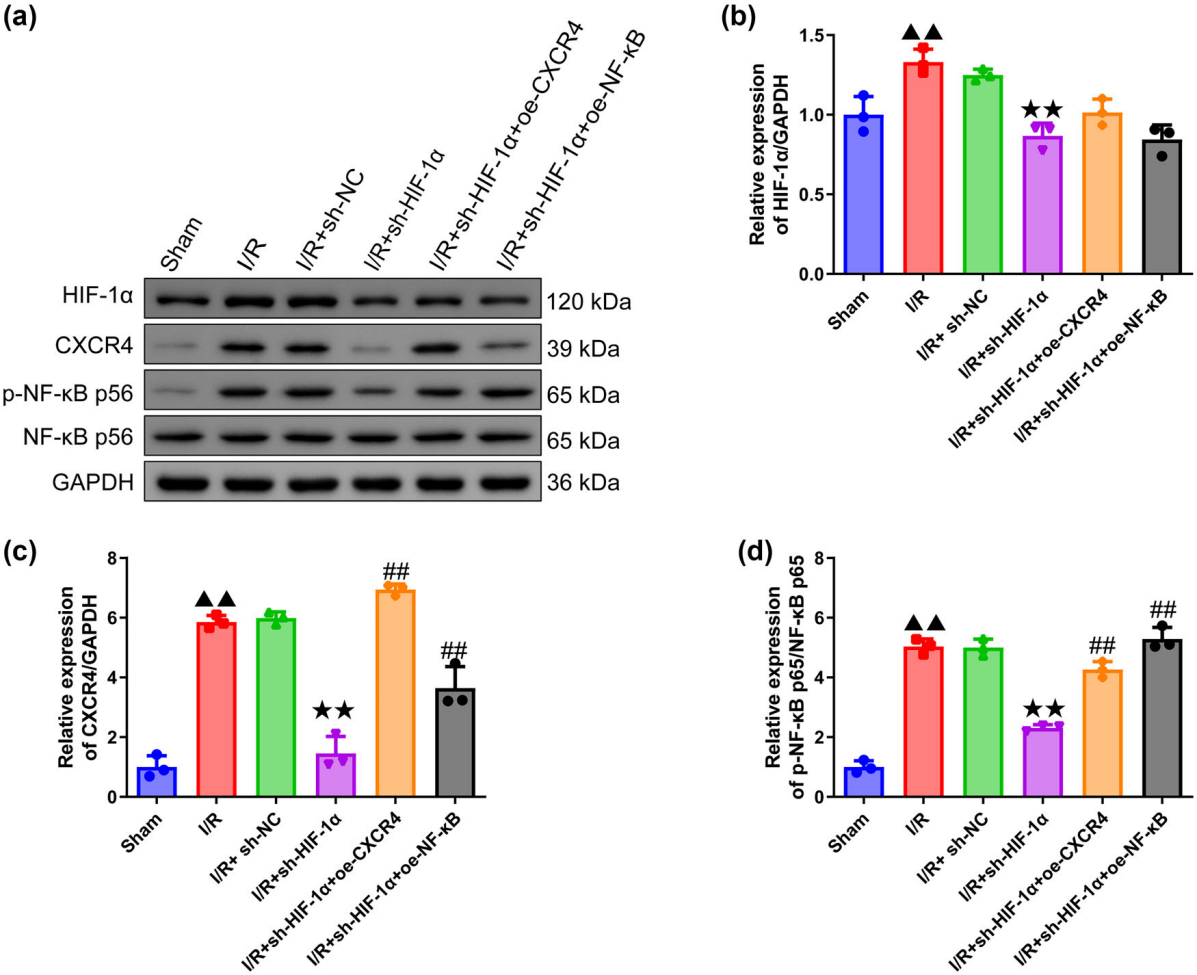
**Quantification on HIF‐1α/CXCR4/NF‐κB axis‐related protein levels in MCAO‐modeled rats. (a)** Representative figures of protein blot from Western blot assay (*n* = 6). **(b–d)** Relative expressions of HIF‐1α **(b)**, CXCR4 **(c)**, and NF‐κB p65 **(d)** in each group of rats were calculated via Western blot (*n* = 6). GAPDH was the housekeeping control. Data were presented as the mean ± standard deviation. ^▲▲^ *p *< .01, vs. Sham. ^★★^ *p *< .01, vs. I/R+sh‐NC. ^#^ *^#^p *< .01, vs. I/R+sh‐HIF‐1α. P‐NF‐κB: phosphorylated‐nuclear factor kappa B.

A oane‐way ANOVA was used to analyze Western blot results (HIF‐1α: df = 5, *F* = 16.828, *p *= .000; CXCR4: df = 5, *F* = 103.151, *p *= .000; p‐NF‐κB p65/NF‐κB p65: df = 5, *F* = 133.131, *p *= .000), followed by Tukey's test (Figure 4a–d). The modeling of MCAO in rats led to the upregulation of HIF‐1α/CXCR4/NF‐κB p65 expression (HIF‐1α, CXCR4, p‐NF‐κB p65/NF‐κB p65 expression in I/R group compared to sham group: *p *= .004/.000/.000) (Figure 4a–d), but the silencing of HIF‐1α could downregulate the expression level of HIF‐1α/CXCR4/NF‐κB p65 (HIF‐1α, CXCR4, p‐NF‐κB p65/NF‐κB p65 expression in I/R+sh‐HIF‐1α group compared to I/R+sh‐NC group: *p *= .001/.000/.000) (Figure 4a–d). Besides, the effects of silencing of HIF‐1α on CXCR4/NF‐κB p65 expression were reversed after the overexpression of CXCR4 and NF‐κB (CXCR4, p‐NF‐κB p65/NF‐κB p65 expression in I/R+sh‐HIF‐1α+oe‐CXCR4 and I/R+sh‐HIF‐1α+oe‐NF‐κB groups compared to I/R+sh‐HIF‐1α group: All *p *= .000) (Figure 4c and d).

### 3.5 Effects of HIF‐1α silencing on cytotoxicity and ROS generation in OGD/R‐treated HT22 cells

**FIGURE 5:**
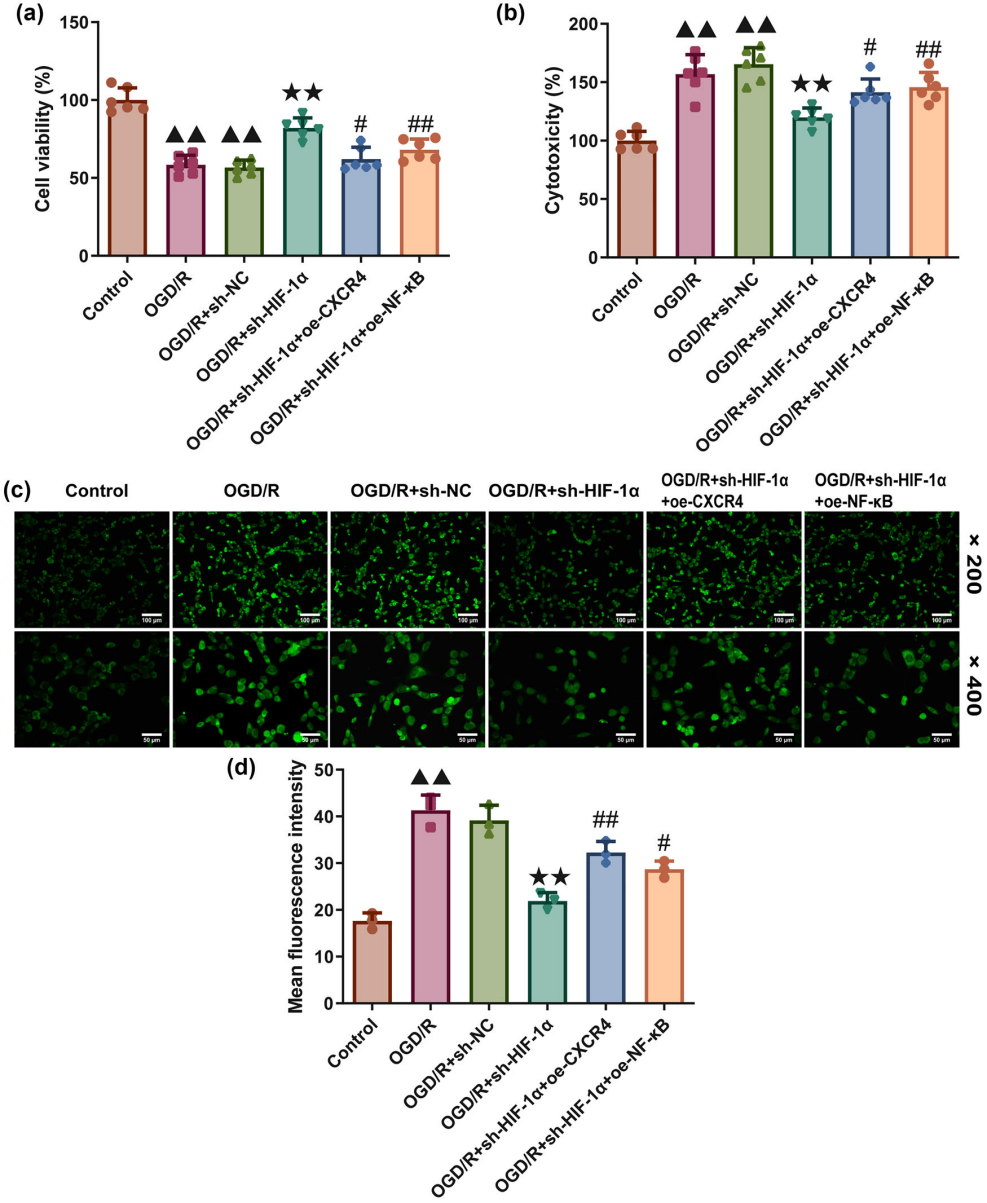
**HIF‐1α knockdown attenuates OGD/R‐induced cytotoxicity and ROS generation in mice hippocampal HT22 cells. (a)** MTT assay was applied to determine the viability of mice hippocampal HT22 cells in each group (*n* = 3). **(b)** LDH assay kit was adopted to quantify the cytotoxicity of mice hippocampal HT22 cells in different groups (*n* = 3). **(c, d)** ROS generation in mice hippocampal HT22 cells of each group was visualized using the DCFH‐DA probe (*n* = 3). Magnification: ×200 and ×400, scale bar: 100 and 50 µm. Data were presented as the mean ± standard deviation. ^▲▲^ *p *< .01, vs. Control. ^★★^ *p *< .01, vs. OGD/R+sh‐NC. ^#^ *p *< .05, ^#^ *^#^p *< .01, vs. I/R+sh‐HIF‐1α. OGD/R: oxygen glucose deprivation/re‐oxygenation; LDH: lactate dehydrogenase; DCFH‐DA: 2′−7′dichlorofluorescin diacetate.

An OGD/R modeling was established in HT22 cells to further evaluate the effects of HIF‐1α silencing on the viability, cytotoxicity, and ROS generation in vitro. A oAne‐way ANOVA was used to analyze results of cell viability, cytotoxicity, and ROS (cell viability: df = 5, *F* = 38.082, *p *= .000; cytotoxicity: df = 5, *F* = 24.963, *p *= .000; ROS: df = 5, *F* = 43.889, *p *= .000). According to Tukey's post hoc test results of MTT assay (Figure 5a), LDH quantification test (Figure 5b), and ROS determination (Figure 5c), compared to control group, the viability of HT22 cells was reduced in response to OGD/R modeling (*p *= .000), with the increased cytotoxicity (*p *= .000) and ROS content (*p *= .000) (Figure 5a–c), while the silencing of HIF‐1α enhanced the viability of modeled cells (*p *= .000) and alleviated the cytotoxicity (*p *= .000) and ROS content (*p *= .000) compared to OGD/R+sh‐NC group (Figure 5a–c). However, the overexpression of CXCR4 and NF‐κB both led to diminished viability and increased cytotoxicity and ROS content in OGD/R‐modeled HT22 cells (OGD/R+sh‐HIF‐1α+oe‐CXCR4 and OGD/R +sh‐HIF‐1α+oe‐NF‐κB groups compared to OGD/R+sh‐HIF‐1α group, cell viability: *p *= .000/.013; cytotoxicity: *p *= .048/.011; ROS: *p *= .002/.048) (Figure 5a–c).

### 3.6 Effects of HIF‐1α silencing on HIF‐1α/CXCR4/NF‐κB p65 expression in OGD/R‐treated HT22 cells

**FIGURE 6:**
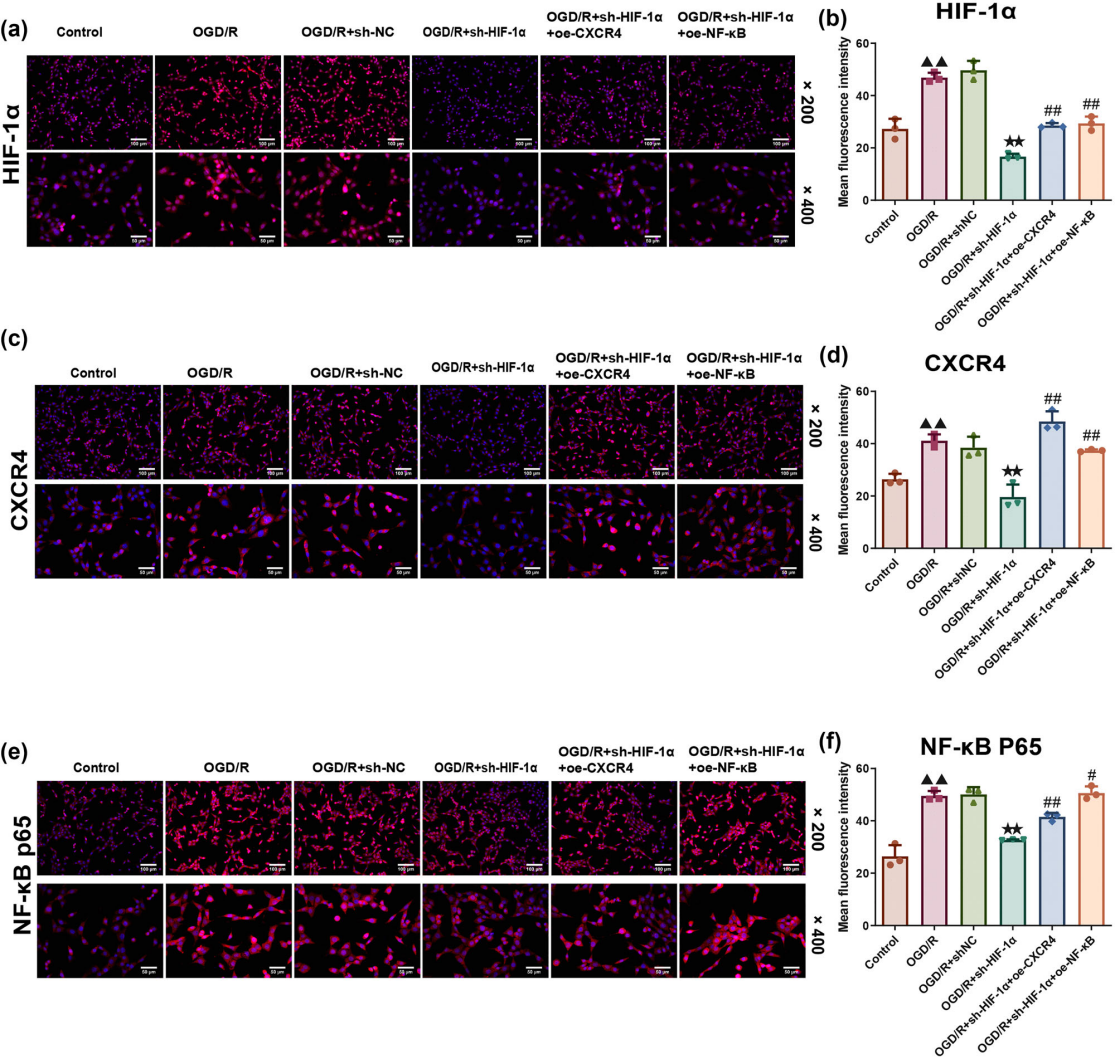
**Quantification on HIF‐1α/CXCR4/NF‐κB expression in OGD/R‐modeled mice hippocampal HT22 cells. (a–f)** Immunofluorescence assay was applied to determine HIF‐1α/CXCR4/NF‐κB expression in OGD/R‐modeled mice hippocampal HT22 cells (*n* = 3). Magnification: ×200 and ×400, scale bar: 100 and 50 µm. Data were presented as the mean ± standard deviation. ^▲▲^ *p *< .01, vs. Control. ^★★^ *p *< .01, vs. OGD/R+sh‐NC. ^#^ *p *< .05, ^#^ *^#^p *< .01, vs. I/R+sh‐HIF‐1α.

A one‐way ANOVA was used to analyze the results of the immunofluorescence assay (HIF‐1α: df = 5, *F* = 51.151, *p *= .000; CXCR4: df = 5, *F* = 35.546, *p *= .000; NF‐κB p65: df = 5, *F* = 21.954, *p *= .000). Based on Tukey's post hoc test results, it could be concluded that the OGD/R modeling caused an upward trend in HIF‐1α/CXCR4/NF‐κB p65 expression, as reflected by the increased fluorescence intensity (compared to control group, HIF‐1α: *p *= .000; CXCR4: *p *= .001; NF‐κB p65: *p *= .000) (Figure 6a–f), while HIF‐1α silencing led to contrary results (compared to OGD/R+sh‐NC group, HIF‐1α: *p *= .000; CXCR4: *p *= .000; NF‐κB p65: *p *= .001) (Figure 6a–f). Besides, overexpression of CXCR4 and NF‐κB could reverse the effects of HIF‐1α silencing in these modeled cells (OGD/R+sh‐HIF‐1α+oe‐CXCR4 and OGD/R+sh‐HIF‐1α+oe‐NF‐κB groups compared to OGD/R+sh‐HIF‐1α group: HIF‐1α: *p *= .005/.003; CXCR4: both *p *= .000; NF‐κB p65: *p *= .041/.001) (Figure 6a–f, *p *< .01).

## 4 DISCUSSION

Research has shown that there is a difference in prognosis and good outcomes between males and females in the clinical treatment of acute ischemic stroke, despite having a higher degree of disability upon admission, female patients are more likely to achieve good functional outcomes upon discharge (Bonkhoff et al., 2021). Therefore, male rats were selected for our study to exclude gender interference. Inflammation and oxidative stress are associated with poststroke induced neuronal and brain tissue damage (Della Corte et al., 2016; Khoshnam et al., 2017). It has been documented that IS, through the production of excessive substances like ROS, leads to oxidative stress, which results in widespread damage to trigger cell death (Woodruff et al., 2011). Cytokines are those crucial players acting actively in the pathophysiologic processes that occur following the onset of IS and are present within the center and periphery of the ischemic focus (Tuttolomondo et al., 2016; Wu et al., 2020). Of these cytokines, TNF‐α, IL‐1β, and IL‐6 have been already discussed, since ROS has been underlined to increase the expression levels of genes encoding these proinflammatory factors (Turovsky et al., 2021; Zafar et al., 2021). Meanwhile, some solid shreds of evidence have suggested that the low blood flow during IS can trigger some reactions producing free radicals (as marked by the elevation of MDA) and damaging DNA (as indicated by the content of 8‐OHdG) (Syafrita et al., 2020). It should be noticed that HIF‐1α, the gene of our interest, is closely associated with ROS production and oxidative stress, despite the debate over its effects on oxidative stress (Qin et al., 2022). Also, another review by Malkov et al. (2021) has systematically addressed the specific effects of HIF‐1α on these cytokines. HIF‐1α can directly regulate the levels of all three cytokines in rheumatoid arthritis synovial fibroblasts (Hu et al., 2016). Also, due to the inhibition of VHL protein, mice with constitutive HIF‐1α expression presented an upward trend in the levels of these cytokines (Shah et al., 2008). Further instances have consolidated that activation of the HIF pathway through mimicking hypoxia can reduce the levels of these inflammatory markers in the murine colitis model (Cummins et al., 2008). Some contradictory results, however, have been proposed while trying to interpret the specific mechanism of HIF‐1α on stroke. For example, the study by Amin et al. (2021) has concluded that HIF‐1α could attenuate the ischemic brain damage of rats, as suggested by the observable increase of HIF‐1 level in the group administrated with the HIF‐1α activator dimethyloxalylglycine (DMOG). Oppositely, suppression of HIF‐1α can worsen the viability, permeability, and apoptosis in OGD/R‐modeled human brain microvascular endothelial cells (hBMECs) (Ni et al., 2022). We here revealed and confirmed that HIF‐1α silencing could attenuate the neuroinflammation and oxidative stress via diminishing the contents of LDH, proinflammatory cytokines as well as the markers MDA and 8‐OHdG. Considering the disparities with regard to the specific effects of HIF‐1α in ischemic injury, future investigation is warranted to elucidate the detailed mechanisms.

The chemokines, meanwhile, also work as crucial cytokines in inflammation during IS (Liu et al., 2018). As to the relevant mechanism implicated, the HIF‐1α‐dependent recruitment of bone marrow‐derived endothelial progenitor cells (bmEPCs) to the ischemic brain was underlined to be associated with C‐X‐C Motif Chemokine Ligand 12 (CXCL12)/CXCR4 axis, for instance (Liu et al., 2018). As a chemokine receptor protein with diverse modulatory effects in both the immune system and neurodevelopment and a major player of the SDF‐1α‐CXCR4/CXCR7 axis, CXCR4 can decide the migration of cells toward the ischemic lesions and act as a potential prognostic indicator of acute IS (Bonham et al., 2018; Gójska‐Grymajło et al., 2018; Huang et al., 2019). Existing studies have addressed that HIF‐1α/CXCR4 can reduce neuronal apoptosis in traumatic brain injury‐modeled rats (Guo et al., 2020) and that both HIF‐1α and CXCR4 suppression can attenuate the development of radiation necrosis in the brains of mice (Yang et al., 2018). AMD3100, the CXCR4 antagonist, can evidently reduce the contents of the inflammatory cytokines in the atria of atrial fibrillation (AF)‐modeled mice (Liu et al., 2021). The data of our current study supported that CXCR4 overexpression could not only aggravate the cerebral inflammation of MCAO‐modeled rats and the cytotoxicity in OGD/R‐modeled HT22 cells but also reversed the effects of HIF‐1α silencing. However, it should be noted that the binding relationship between CXCR4 and HIF‐1α has not been validated in our study, rendering a limitation.

Some prior evidence has recognized the NF‐κB pathway as the downstream effector while trying to interpret the specific mechanism of stroke (Chen et al., 2022; Viswanatha et al., 2021). NF‐κB pathway has been recognized as a crucial modulator related to the inflammatory process in IS, which can regulate the expression of both proinflammatory and proapoptotic genes (Crack & Taylor, 2005; Howell & Bidwell, 2020). While linking HIF‐1α and NF‐κB, it has been illustrated that ischemic preconditioning may upregulate HIF‐1α expression in pyramidal neurons of CA1 region (CA1), which then enhances NF‐κB activation (Lee et al., 2017). In addition, NF‐κB is targeted by CXCR4 in early brain injury following SAH (Liu et al., 2023). When it comes to our study, relevant data have supported that HIF‐1α silencing could attenuate the phosphorylation of NF‐κB p65 in MCAO‐modeled male rats and the fluorescence activity of NF‐κB p65 in OGD/R‐modeled HT22 cells. Oppositely, such effects of HIF‐1α silencing were then confirmed to be reversed upon the overexpression of NF‐κB p65. These findings hinted that the alleviative effects of HIF‐1α knockdown on oxidative stress and inflammation in IS were achieved via the NF‐κB pathway. Additionally, it is interesting that the expression trend of HIF‐1a is consistent in MACO female and male mice (Limatola et al., 2010), and the NF‐κB related pathway is predicted to be involved in the occurrence of ischemic stroke in women (Xie et al., 2020), suggesting that the results of our study may be generalizable to female and require further exploration.

Collectively speaking, this study showed that HIF‐1α silencing could attenuate the inflammation and oxidative stress in IS based on the animal MCAO model and the cell OGD/R model, which was possibly associated with the CXCR4/NF‐κB pathway. These discoveries, we hope, may provide novel insights for the management of IS and other ischemic diseases.

## AUTHOR CONTRIBUTIONS

**Gao Chen**: Conceptualization; writing—review and editing; methodology. **Xi Wang**: Data curation. **Zhan Jin**: Funding acquisition; investigation; formal analysis. **Gao‐Bo Hu**: Project administration; resources; writing—original draft. **Qi‐Hui Yu**: Software; supervision; validation. **Hai‐Yan Jiang**: Visualization.

## FUNDING

This study was supported the Quzhou City Science and Technology Research Project [2022K111].

## CONFLICT OF INTEREST STATEMENT

The authors declare no conflicts of interest

### PEER REVIEW

The peer review history for this article is available at https://publons.com/publon/10.1002/brb3.70039.

## Data Availability

The datasets generated during and/or analyzed during the current study are available from the corresponding author on reasonable request.
